# The ability of biomarkers to assess the severity of atopic dermatitis

**DOI:** 10.1016/j.jacig.2023.100175

**Published:** 2023-09-27

**Authors:** Takeshi Nakahara, Daisuke Onozuka, Satoshi Nunomura, Hidehisa Saeki, Motoi Takenaka, Mai Matsumoto, Yoko Kataoka, Rai Fujimoto, Sakae Kaneko, Eishin Morita, Akio Tanaka, Ryo Saito, Tatsuro Okano, Tomomitsu Miyagaki, Natsuko Aoki, Kimiko Nakajima, Susumu Ichiyama, Makiko Kido-Nakahara, Kyoko Tonomura, Yukinobu Nakagawa, Risa Tamagawa-Mineoka, Koji Masuda, Takuya Takeichi, Masashi Akiyama, Yozo Ishiuji, Michie Katsuta, Yuki Kinoshita, Chiharu Tateishi, Aya Yamamoto, Akimichi Morita, Haruna Matsuda-Hirose, Yutaka Hatano, Hiroshi Kawasaki, Ayano Fukushima-Nomura, Mamitaro Ohtsuki, Koji Kamiya, Yudai Kabata, Riichiro Abe, Hiroshi Mitsui, Tatsuyoshi Kawamura, Gaku Tsuji, Norito Katoh, Masutaka Furue, Kenji Izuhara

**Affiliations:** a Department of Dermatology, Graduate School of Medical Sciences, Kyushu University, Fukuoka, Japan; b Department of Oral Microbe Control, Graduate School of Medicine, Osaka University, Osaka, Japan; k Department of Dermatology, Course of Integrated Medicine, Graduate School of Medicine, Osaka University, Osaka, Japan; c Division of Medical Biochemistry, Department of Biomolecular Sciences, Saga Medical School, Saga, Japan; d Department of Dermatology, Nippon Medical School, Tokyo, Japan; e Department of Dermatology, Graduate School of Biomedical Sciences, Nagasaki University, Nagasaki, Japan; f Department of Dermatology, Osaka Habikino Medical Center, Osaka, Japan; g Department of Dermatology, Shimane University Faculty of Medicine, Shimane, Japan; h Department of Dermatology, Graduate School of Biomedical and Health Sciences, Hiroshima University, Hiroshima, Japan; i Department of Dermatology, St. Marianna University School of Medicine, Kanagawa, Japan; j Department of Dermatology, Kochi Medical School, Kochi University, Kochi, Japan; l Department of Dermatology, Graduate School of Medical Science, Kyoto Prefectural University of Medicine, Kyoto, Japan; m Department of Dermatology, Nagoya University Graduate School of Medicine, Aichi, Japan; n Department of Dermatology, The Jikei University School of Medicine, Tokyo, Japan; o Department of Dermatology, Osaka Metropolitan University Graduate School of Medicine, Osaka, Japan; p Department of Geriatric and Environmental Dermatology, Nagoya City University Graduate School of Medical Sciences, Aichi, Japan; q Department of Dermatology, Faculty of Medicine, Oita University, Oita, Japan; r Department of Dermatology, School of Medicine, Keio University, Tokyo, Japan; s Department of Dermatology, Jichi Medical University, Tochigi, Japan; t Division of Dermatology, Niigata University Graduate School of Medical and Dental Sciences, Niigata, Japan; u Department of Dermatology, Faculty of Medicine, University of Yamanashi, Yamanashi, Japan

**Keywords:** Atopic dermatitis, biomarker, B-PAD, Biomarkers to Predict Clinical Improvement of AD in Patients Treated With Dupilumab, EASI, eotaxin-3, LDH, POEM, pruritus-NRS, precision medicine, SCCA2

## Abstract

**Background:**

To develop precision medicine for atopic dermatitis (AD), it is critical to establish relevant biomarkers. However, the characteristics of various biomarkers have not been fully understood. We previously carried out the Biomarkers to Predict Clinical Improvement of AD in Patients Treated with Dupilumab (B-PAD) study, a comprehensive nationwide study in Japan, to explore biomarkers for AD.

**Objective:**

The aim of this study is to find biomarkers associated with objective and subjective clinical findings in patients with moderate-to-severe AD based on the B-PAD study and to identify biomarkers sensitive enough to assess the severity of AD.

**Methods:**

We performed the B-PAD study as a consortium composed of 19 medical facilities in Japan, enrolling 110 patients with moderate-to-severe AD. We evaluated the Eczema Area and Severity Index (EASI) for objective assessment as well as the Patient-Oriented Eczema Measure (POEM) and a numeric rating scale for pruritus (pruritis-NRS) for subjective assessment, measuring 19 biomarkers at baseline.

**Results:**

We found that 12, 6, and 7 biomarkers showed significant and positive associations with the EASI, POEM, and pruritis-NRS, respectively. Most of the biomarkers associated with either the POEM or the pruritis-NRS were included among the biomarkers associated with EASI. Of the biomarkers examined, CCL26/eotaxin-3 and SCCA2 were the most capable of assessing severity for EASI, as shown by the 2 kinds of receiver operating characteristic analyses, respectively, whereas lactate dehydrogenase was the best for both the POEM and pruritis-NRS, again using the 2 analyses.

**Conclusion:**

We found biomarkers associated with the EASI, POEM, and pruritis-NRS, respectively, based on the B-PAD study. Moreover, we identified CCL26/eotaxin-3 and/or SCCA2 as the biomarkers having the greatest ability to assess severity in the EASI; lactate dehydrogenase did the same for the POEM and pruritis-NRS. These findings will be useful in treating patients with moderate-to-severe AD.

## Introduction

Atopic dermatitis (AD) is a chronic relapsing skin disease accompanied by recurrent itching.^1^ AD is highly heterogenous, with backgrounds of various genetic environmental factors.^2^ Because several novel molecularly targeted drugs have recently become available, treatment for AD has been moving from the “one drug fits all” approach to an approach that is “patient endotype–specific.”^3^

To develop precision medicine for AD, establishment of relevant biomarkers is essential. Many biomarkers, mainly reflecting the type 2 inflammation dominant in the pathogenesis of AD, have been listed; some of them are in use for routine treatments of AD.^4^ However, the comprehensive characteristics of these biomarkers depending on their purposes––assessing risk of susceptibility, severity, and prognosis, as well as predicting drug efficacy and possible side effects—have not been sufficiently well understood to make comparisons among them.

We previously explored such biomarkers in a broad-based study titled Biomarkers to Predict Clinical Improvement of AD in Patients Treated with Dupilumab (B-PAD).^5^^,^^6^ To do so, we assembled a consortium of 19 medical facilities in Japan, all of which were actively grappling with treatments for patients with AD, and enrolled 110 patients with moderate-to-severe AD. We evaluated objective clinical findings according to the Eczema Area and Severity Index (EASI) and subjective clinical findings by 2 different parameters, the Patient-Oriented Eczema Measure (POEM) and the numeric rating scale for pruritus (pruritus-NRS). We measured 19 biomarkers at baseline and regularly after the start of dupilumab injection.

In this investigation, we looked for biomarkers associated with the EASI, POEM, and pruritus-NRS, in patients with moderate-to-severe AD by using the B-PAD study. Moreover, we compared the abilities of the biomarkers that turned out to be associated with the EASI, POEM, and pruritus-NRS to assess severity, and we identified those biomarkers that do it best.

## Results and discussion

**Fig 1:**
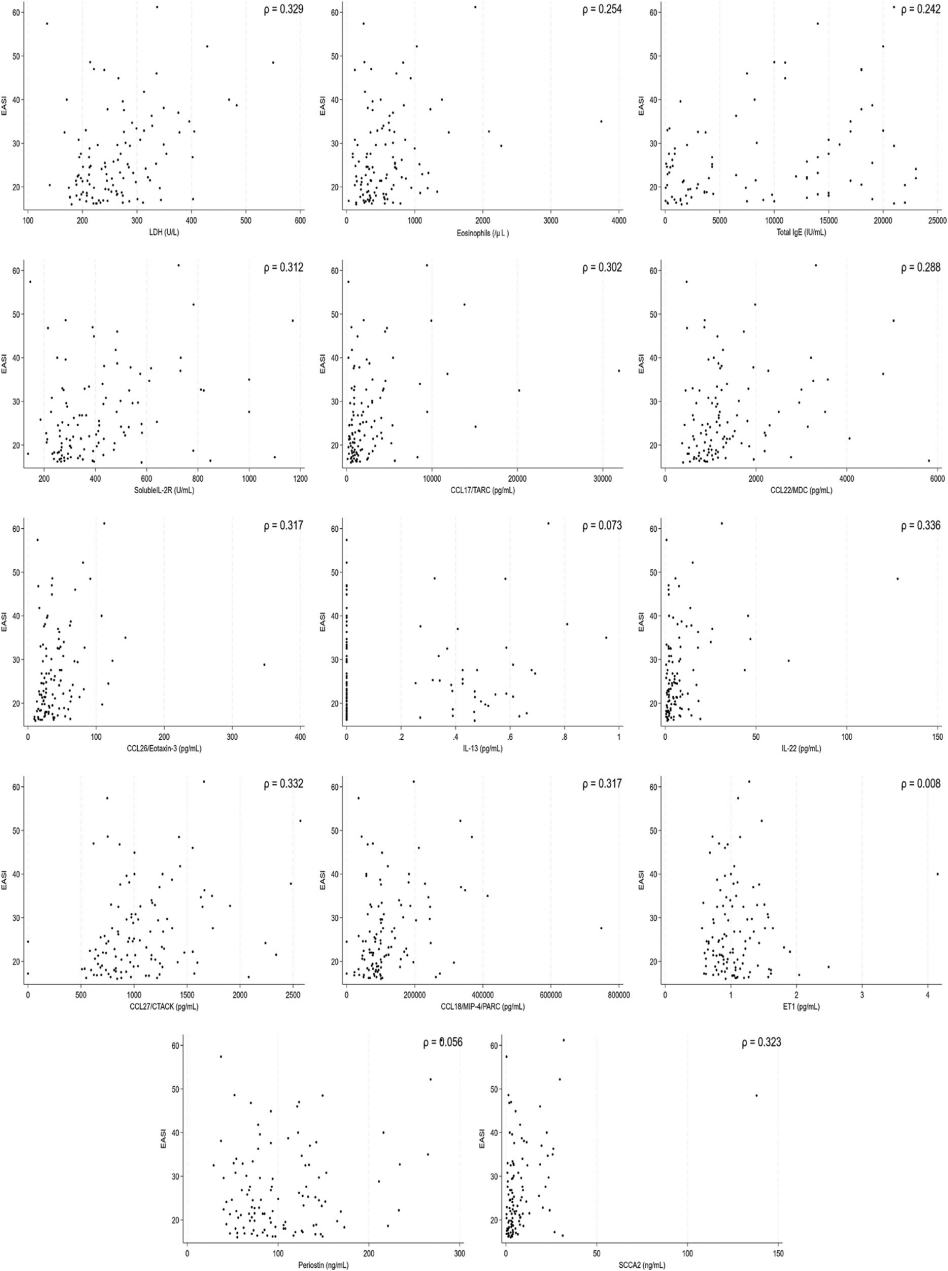
Association of 14 biomarkers with the EASI. Dot plot analysis of the correlation between each biomarker and EASI in the subjects (N = 110) is depicted.

**Fig 2:**
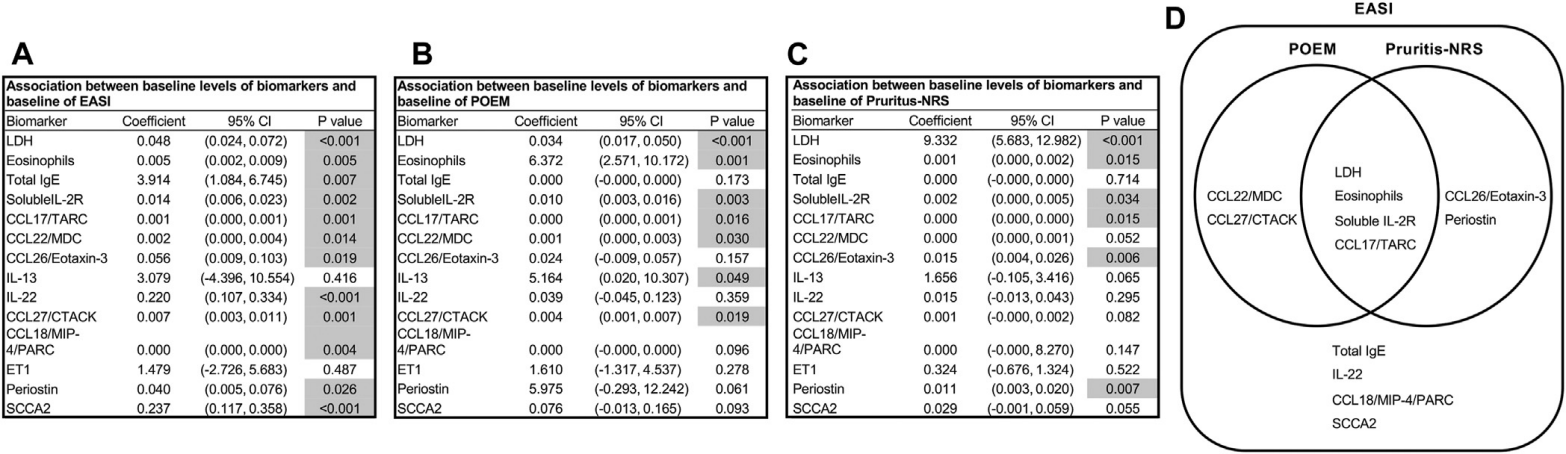
Summary of the associations of 14 biomarkers with clinical findings. Summaries of the associations of each biomarker with EASI (**A**), the POEM (**B**), and the pruritis-NRS (**C**) are depicted. Biomarkers highlighted in gray show significant statistical difference. (**D**) The relationships of the biomarkers associated with the EASI that are also associated with the POEM or the pruritis-NRS are depicted.

**Fig 3:**
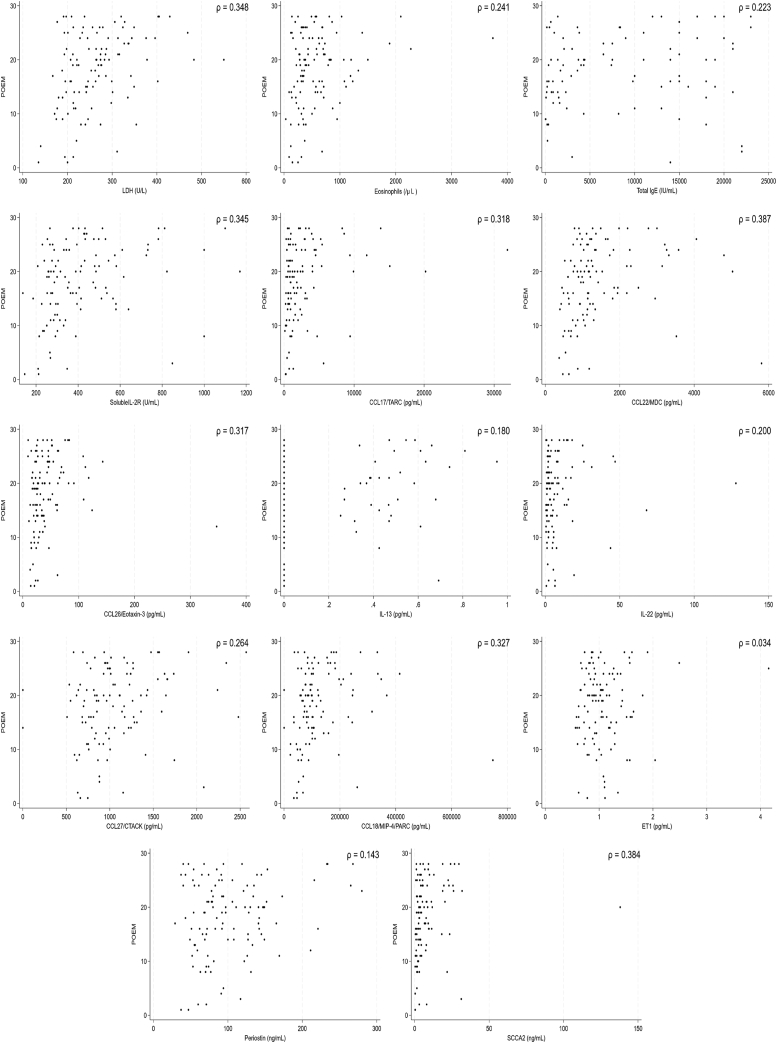
Association of 14 biomarkers with the POEM. Dot plot analysis of the correlation between each biomarker and POEM in the subjects (N = 110) is depicted.

**Fig 4:**
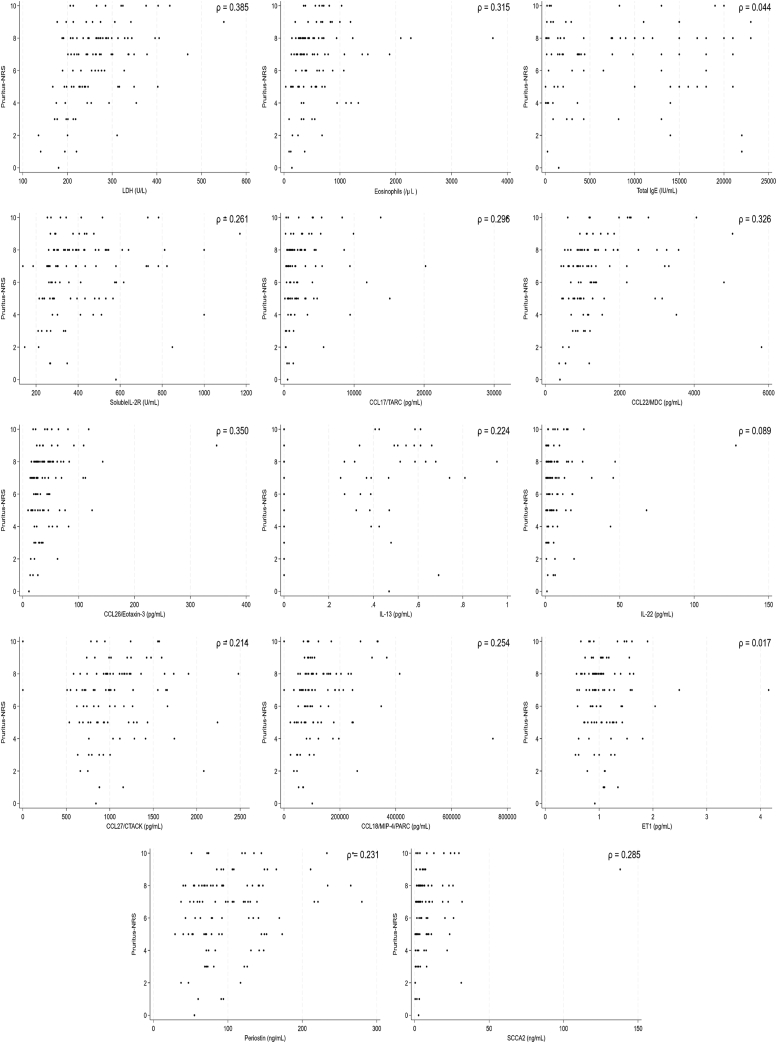
Association of 14 biomarkers with the pruritis-NRS. Dot plot analysis of the correlation between each biomarker and the pruritis-NRS in the subjects (N = 110) is depicted.

The backgrounds and overall clinical findings for the subjects, as well as the biomarkers in the B-PAD study, are described in the Online Repository (available at www.jaci-global.org). We first examined associations between levels of 19 biomarkers and 3 clinical assessments—the EASI, POEM, and pruritis-NRS—before administration of dupilumab. We set the values below the lower limits often observed in IL-24, IL-25, IL-31, IL-33, and TSLP, which are shown in the Online Repository to be zero. Linear regression analyses showed that 12 biomarkers—lactase dehydrogenase (LDH), eosinophils, total IgE, soluble IL-2R, CCL17/TARC, CCL22/MDC, CCL26/eotaxin-3, IL-22, CCL27/CTACK, CCL18/MIP-4/PARC, periostin, and SCCA2—were significantly and positively correlated with the EASI, whereas IL-13 and ET1 were not associated with the EASI (Figs 1 and 2, *A*). As for the POEM, 7 biomarkers—LDH, eosinophils, soluble IL-2R, CCL17/TARC, CCL22/MDC, IL-13, and CCL27/CTACK—showed significant and positive associations (Figs 2, *B* and 3). Moreover, as for the pruritis-NRS, 6 biomarkers—LDH, eosinophils, soluble IL-2R, CCL17/TARC, CCL26/eotaxin-3, and periostin—showed significant and positive associations (Figs 2, *C* and 4). These results suggest that relevant biomarkers are different depending on the clinical outcomes; however, all biomarkers associated with either the POEM or the pruritis-NRS, with the exception of IL-13 for the POEM, are included in the biomarkers associated with the EASI (Fig 2, *D*). Therefore, the 12 biomarkers associated with the EASI are categorized into 4 groups (Fig 2, *D*): (1) biomarkers associated with the EASI, the POEM, and the pruritis-NRS (ie, LDH, eosinophils, soluble IL-2R, and CCL17/TARC); (2) biomarkers associated with the EASI and the POEM but not with the pruritis-NRS (ie, CCL22/MDC and CCL27/CTACK); (3) biomarkers associated with the EASI and the pruritis-NRS but not with the POEM (ie, CCL26/eotaxin-3 and periostin); and (4) biomarkers associated with the EASI only and not with the POEM or the pruritis-NRS (ie, total IgE, IL-22, CCL18/MIP-4/PARC, and SCCA2).

**Fig 5:**
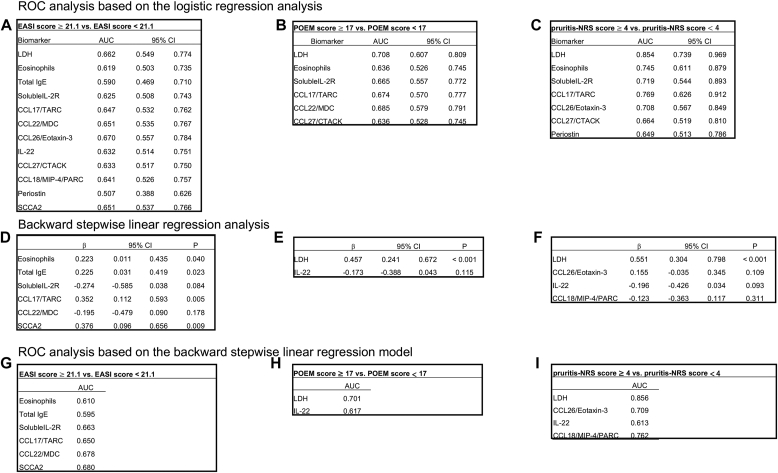
Abilities of the biomarkers to assess clinical severity of AD. The abilities of the biomarkers associated with the EASI (**A** and **D**), the POEM (**B** and **E**), and the pruritis-NRS (**C** and **F**) to assess the severity of each clinical finding by the ROC analysis based on the logistic regression analysis (**A-C**) or by backward stepwise linear regression analysis (**D-F**). **G-I,** AUCs according to the ROC analysis based on the backward stepwise linear regression model, using the data from the backward stepwise linear regression analysis (**D-F**).

We next compared the ability of each biomarker to assess clinical severity in the EASI, POEM, and pruritis-NRS by using 2 methods: receiver operating characteristic (ROC) analyses based on logistic regression analysis and on backward stepwise linear regression model. Compared with the simple ROC analysis based on the logistic regression analysis, the ROC analysis based on the stepwise regression model is useful to find the best combination of independent variables, simplify the model, reduce overfitting, and improve prediction accuracy.^7^ For the ROC analyses, we divided the subjects into 2 groups, severe and nonsevere, for 3 clinical findings**—**EASI score of 21.1 or higher versus less than 21.1, POEM score of 17 or higher versus less than 17, and pruritis-NRS score of 4 or higher versus less than 4. next, we examined the abilities of the biomarkers identified in Fig 2, *A*-*C* to assess the severities of each clinical finding by the ROC analysis based on the logistic regression analysis. A total of 12 biomarkers showed areas under the curve (AUCs) of 0.507 to 0.670, with the highest AUC value found in the case of CCL26/eotaxin-3 for the EASI; 6 biomarkers showed AUCs of 0.636 to 0.708, with the highest AUC value found in the case of LDH for the POEM; and 7 biomarkers showed AUCs of 0.649 to 0.854, with the highest AUC value found in the case of LDH for the pruritis-NRS (Fig 5, *A*-*C*). We then found that 6, 2, and 4 biomarkers are associated with the EASI, POEM, and pruritis-NRS, respectively, according to backward stepwise linear regression analysis (Fig 5, *D*-*F*). SCCA2 showed the highest association for the EASI (β = 0.376; *P* = .009), and LDH showed the highest associations for the POEM (β = 0.457; *P* < .001) and the pruritis-NRS (β = 0.551; *P* < .001). The ROC analysis based on the backward stepwise linear regression model, using the same definitions of the severe and nonsevere groups, as Fig 5, *A-C*, shows AUC values of 0.680 for SCCA2 for the EASI, 0.701 for LDH for the POEM, and 0.856 for LDH for the pruritis-NRS (Fig 5, *G*-*I*). No other combination of the other biomarkers with SCCA2 or LDH significantly upregulated the AUCs compared with either SCCA2 or LDH alone (the AUCs for the EASI ranged from 0.642 to 0.680, the AUC for the POEM was 0.710, and the AUCs for the pruritis-NRS ranged from 0.858 to 0.877 [described in the Online Repository]). These results demonstrate that CCL26/eotaxin-3 and/or SCCA2 can assess severity of AD according to the EASI most sensitively, whereas LDH is most sensitive biomarker for the POEM and the pruritis-NRS.

It is of great interest that (1) most of the biomarkers associated with the POEM or the pruritis-NRS are included among those of the EASI and (2) the biomarkers associated with the POEM or the pruritis-NRS overlap, although there are distinct biomarkers associated with either finding. These results would suggest that the EASI, which is the representative evaluation method yielding objective findings for patients with AD, reflects well the subjective findings for patients with AD evaluated by the POEM or the pruritis-NRS. The results also suggest that 2 methods yielding subjective findings, namely, the POEM and the pruritis-NRS, reflect clinical outcomes of patients with AD common in some part but distinct in some part. CCL26/eotaxin-3, a member of the CC chemokine family, is known to be produced in dermal fibroblasts and endothelial cells by stimulation of IL-4 and IL-13.^8^ It has been previously shown that CCL26/eotaxin-3 levels in blood are elevated in both infant and adult patients with AD.^9^^,^^10^ SCCA2, a member of the ovalbumin serpin/clade B serpin family, is a downstream molecule of IL-4 and IL-13 produced in keratinocytes.^11^ It has been shown that serum SCCA2 levels are upregulated in both adult and child patients with AD according to clinical severity as well.^12^^,^^13^ Thus, CCL26/eotaxin-3 and SCCA2, the biomarkers that are most capable of assessing severity in the EASI, are surrogate biomarkers of IL-4 and IL-13, which are signature cytokines of type 2 inflammation. In contrast, LDH is a long-known biomarker for general inflammation^4^ and its levels are also elevated in patients with AD.^14^ It is of note that nevertheless, LDH has higher abilities to assess severity based on the POEM and the pruritis-NRS than various type 2 biomarkers are.

One limitation of this investigation is that the subjects were limited to patients with moderate-to-severe AD and did not include patients with mild AD, because the B-PAD study did not include such patients. Therefore, no information about them is shown in the present study. Further studies covering patients with mild AD are needed. Another limitation is that we could not find a combination of biomarkers with SCCA2 or LDH to enhance the abilities to diagnose severity of the EASI, POEM, and pruritis-NRS. It may be due to our having investigated only biomarkers having similar characteristics. Further studies including other biomarkers that are assumed to have characteristics different from those of the listed biomarkers with SCCA2 or LDH are awaited.

In conclusion, we identified biomarkers associated with the EASI, POEM, and pruritis-NRS, respectively, based on the B-PAD study, which was a comprehensive nationwide study in Japan to explore biomarkers for AD. Moreover, we found that CCL26/eotaxin-3 and/or SCCA2 has the greatest ability to assess severity of AD according to the EASI, whereas LDH does so on the basis of the POEM and pruritis-NRS. The present findings will be of great use in developing treatments for patients with moderate-to-severe AD; moreover, it gives us clues to clarify the pathogenesis behind moderate-to-severe AD.

## Disclosure statement

Supported by Sanofi and 10.13039/100009857Regeneron (grant SGZ-2018-11996).

Disclosure of potential conflict of interest: T. Nakahara has received lecture fees and/or research funds from 10.13039/501100012351Mitsubishi Tanabe Pharma, 10.13039/100009954Taiho Pharmaceutical, Torii Pharmaceutical, Maruho, 10.13039/100004339Sanofi, 10.13039/100006483AbbVie, Eli Lilly Japan, and 10.13039/501100013671Sun Pharma. H. Saeki has received lecture fees, research funds, or scholarship donations from 10.13039/501100012351Mitsubishi Tanabe Pharma, 10.13039/100009954Taiho Pharmaceutical, Torii Pharmaceutical, Maruho, 10.13039/501100004095Kyowa Kirin, 10.13039/100004339Sanofi, 10.13039/100006483AbbVie, 10.13039/100008792Novartis Pharma, Eli Lilly Japan, Kyorin Pharmaceutical, 10.13039/501100003769Eisai, Tokiwa Pharmaceutical, Japan Tobacco, and LEO Pharma. Y. Kataoka has received lecture honoraria from 10.13039/100004339Sanofi, Pfizer, and AbbVie, as well as research funding from 10.13039/100004339Sanofi, Leo Pharma, 10.13039/100004319Pfizer, Maruho, Eli Lilly, 10.13039/100006483AbbVie, and Otsuka. S. Kaneko has received grants as an investigator, as well as honoraria as a speaker from 10.13039/100014422Eli Lilly Japan. A. Tanaka has received honoraria from Eli Lilly, Kaken Seiyaku, 10.13039/100004339Sanofi, 10.13039/100009954Taiho Pharma, AbbVie, Kyorin Pharmaceutical, Mitsubishi-Tanabe, Torii Pharmaceutical, 10.13039/100004319Pfizer, and Maruho as a speaker, as well as research grants from Eli Lilly, Sanofi, Teijin Pharma, Taiho Pharma, Mitsubishi-Tanabe, Torii Pharmaceutical, and Maruho. R. Tamagawa-Mineoka has received research grants from Maruho and Mitsubishi Tanabe Pharma. K. Masuda has received honoraria as a speaker for Sanofi and grants as an investigator for Eli Lilly Japan. Takuya Takeichi has received grants paid to his institution (Nagoya University) from Boehringer Ingelheim and lecture fees from Sanofi. M. Akiyama has received research support from Novartis and Boehringer Ingelheim; personal fees from Maruho and Sanofi; and grants paid to his institution (Nagoya University) from Tanabe-Mitsubishi, Taiho, AbbVie, Maruho, Ono, and Sun Pharma. Y. Ishiuji has received honoraria as a speaker from Maruho, Sanofi, and AbbVie. M. Katsuta has received honoraria as a speaker from Janssen Pharma. Y. Hatano has received honoraria as well as consultancies to sponsoring organizations from Sanofi, TAIHO, Janssen Pharma, Maruho, Pfizer Japan, Sun Pharma Japan, Torii Pharmaceutical, AbbVie, KAKEN, and UCB Japan. N. Katoh has received honoraria as a speaker/consultant for Sanofi, Maruho, AbbVie, Eli Lilly Japan, and Leo Pharma and has received grants as an investigator from Maruho, Eli Lilly Japan, Sun Pharma, Taiho Pharmaceutical, Torii Pharmaceutical, Boehringer Ingelheim Japan, and Leo Pharma. K. Izuhara has received grants from Shino-test Co Ltd and Torii Pharmaceutical, personal fees from Shino-test Co Ltd, and speaker fees from Sanofi, Maruho, and Leo Pharma. The rest of the authors declare that they have no relevant conflicts of interest.

## References

[1] Furue M., Chiba T., Tsuji G., Ulzii D., Kido-Nakahara M., Nakahara T. (2017). Atopic dermatitis: immune deviation, barrier dysfunction, IgE autoreactivity and new therapies. Allergol Int.

[2] Bieber T., Traidl-Hoffmann C., Schappi G., Lauener R., Akdis C., Schmid-Grendlmeier P. (2020). Unraveling the complexity of atopic dermatitis: the CK-CARE approach toward precision medicine. Allergy.

[3] Bakker D., de Bruin-Weller M., Drylewicz J., van Wijk F., Thijs J. (2023). Biomarkers in atopic dermatitis. J Allergy Clin Immunol.

[4] Renert-Yuval Y., Thyssen J.P., Bissonnette R., Bieber T., Kabashima K., Hijnen D. (2021). Biomarkers in atopic dermatitis-a review on behalf of the International Eczema Council. J Allergy Clin Immunol.

[5] Nakahara T., Izuhara K., Onozuka D., Saeki H., Nunomura S., Takenaka M. (2023). Exploring biomarkers to predict clinical improvement of atopic dermatitis in patients treated with dupilumab (B-PAD study). Clin Exp Allergy.

[6] Nakahara T., Izuhara K., Onozuka D., Nunomura S., Tamagawa-Mineoka R., Masuda K. (2020). Exploration of biomarkers to predict clinical improvement of atopic dermatitis in patients treated with dupilumab: a study protocol. Medicine (Baltimore).

[7] Moons K.G.M., Donders A., Rogier T., Steyerberg E.W., Harrell F.E. (2004). Penalized maximum likelihood estimation to directly adjust diagnostic and prognostic prediction models for overoptimism: a clinical example. J Clin Epidemiol.

[8] Ahmadi Z., Hassanshahi G., Khorramdelazad H., Zainodini N., Koochakzadeh L. (2016). An overlook to the characteristics and roles played by eotaxin network in the pathophysiology of food allergies: allergic asthma and atopic dermatitis. Inflammation.

[9] Kagami S., Kakinuma T., Saeki H., Tsunemi Y., Fujita H., Nakamura K. (2003). Significant elevation of serum levels of eotaxin-3/CCL26, but not of eotaxin-2/CCL24, in patients with atopic dermatitis: serum eotaxin-3/CCL26 levels reflect the disease activity of atopic dermatitis. Clin Exp Immunol.

[10] McAleer M.A., Jakasa I., Stefanovic N., McLean W.H.I., Kezic S., Irvine A.D. (2021). Topical corticosteroids normalize both skin and systemic inflammatory markers in infant atopic dermatitis. Br J Dermatol.

[11] Izuhara K., Yamaguchi Y., Ohta S., Nunomura S., Nanri Y., Azuma Y. (2018). Squamous cell carcinoma antigen 2 (SCCA2, SERPINB4): an emerging biomarker for skin inflammatory diseases. Int J Mol Sci.

[12] Okawa T., Yamaguchi Y., Kou K., Ono J., Azuma Y., Komitsu N. (2018). Serum levels of squamous cell carcinoma antigens 1 and 2 reflect disease severity and clinical type of atopic dermatitis in adult patients. Allergol Int.

[13] Nagao M., Inagaki S., Kawano T., Azuma Y., Nomura N., Noguchi Y. (2018). SCCA2 is a reliable biomarker for evaluating pediatric atopic dermatitis. J Allergy Clin Immunol.

[14] Kou K., Aihara M., Matsunaga T., Chen H., Taguri M., Morita S. (2012). Association of serum interleukin-18 and other biomarkers with disease severity in adults with atopic dermatitis. Arch Dermatol Res.

