# Supplementary material for: The ability of biomarkers to assess the severity of atopic dermatitis
Source: J Allergy Clin Immunol Glob. 2023 Sep 27;3(1):100175. doi: 10.1016/j.jacig.2023.100175 (PMC10616407; doi:10.1016/j.jacig.2023.100175)
Supplement: Supplementary data [file mmc1.docx]

**ONLINE REPOSITORY**

**Patients**

This is a post-hoc analysis of the B-PAD study aiming at exploring biomarkers to predict clinical improvement of atopic dermatitis in patients treated with dupilumab [1]. We made a consortium composed of 19 medical facilities in Japan (Kyushu University, Fukuoka, Nippon Medical School, Tokyo, Nagasaki University, Nagasaki, Osaka Habikino Medical Center, Osaka, Shimane University, Shimane, Hiroshima University, Hiroshima, St. Marianna University, Kanagawa, Kochi Medical School, Kochi, Osaka University, Osaka, Kyoto Prefectural University of Medicine, Kyoto, Nagoya University, Aichi, The Jikei University School of Medicine, Tokyo, Osaka Metropolitan University, Osaka, Nagoya City University, Aichi, Oita University, Oita, Keio University, Tokyo, Jichi Medical University, Tochigi, Niigata University, Niigata, University of Yamanashi, Yamanashi) for the B-PAD study. We enrolled 110 (74 male) Japanese patients with moderate-to-severe atopic dermatitis (AD) between 10 October 2019 and 31 March 2022. Sample collection and all procedures were approved by the Ethics Committee of each medical facility in accordance with the ethical standards of the Helsinki Declaration of 1975. Informed consent was obtained from all patients.

All patients were concordant with the Japanese Guidelines for AD [2]. They fulfilled the criteria that the age is not less than 18 years, Eczema Area and Severity Index (EASI) is not less than 16, Investigator's Global Assessment (IGA) is not less than three, the body surface area is not less than 10%, and they had suffered chronic AD for at least three years before the start of this study. The patients were required to discontinue cyclosporine, oral steroids, or phototherapy at least 4 weeks prior to the collection of the samples.

Objective clinical findings were evaluated by EASI. Subjective symptoms were assessed by Patient-Oriented Eczema Measure (POEM) and Numerical Rating Scale for pruritus (pruritus-NRS). The basic characteristics of the examined subjects are described in Table S1.

**Measurements of biomarkers**

We measured 19 biomarkers (LDH (lactase dehydrogenase), blood eosinophils, total IgE, soluble interleukin-2 receptor (IL-2R), CC chemokine ligand (CCL)17/thymus and activation-regulated chemokine (TARC), CCL22/MDC, CCL26/eotaxin-3, IL-13, IL-22, IL-31, CCL27/CTACK, CCL18/MIP-4/PARC, IL-24, IL-25, IL-33, thymic stromal lymphopoietin (TSLP), endothelin-1 (ET-1), periostin, and squamous cell carcinoma antigen 2 (SCCA2)). We ordered the measurements of LDH, blood eosinophils, total IgE, soluble IL-2R, and CCL17/TARC to LSI Medience Co. Ltd (Tokyo, Japan), those of periostin and SCCA2 to Shino-test Co. Ltd (Sagamihara, Japan) and those of the other biomarkers to Kyushu Prosearch LLP (Fukuoka, Japan). We deleted from the analyses the samples showing less than the lower limits of IL-13 (0.240 pg/mL), IL-31 (0.948 pg/mL), IL-24 (15.6 pg/mL), IL-25 (125 pg/mL), IL-33 (3.13 pg/mL), and TSLP (31.3L pg/mL), and the samples showing more than the upper limit of total IgE (25,000 IU/mL). Many samples for IL-24, IL-25, IL-31, IL-33, and TSLP showed below the lower limits shown in Fig S1-3. The overall results of the biomarkers in the subjects are described in Table S1.

**Statistical analysis**

We used backward stepwise linear regression models to investigate the associations of each biomarker with EASI, POEM, and pruritus-NRS. We defined the severe and non-severe groups of the subjects as ≥ 21.1 or < 21.1 for EASI, ≥ 17 or < 17 for POEM, and ≥ 4 or < 4 for pruritus-NRS, respectively. The area under the receiver operating characteristic (ROC) curve (AUC) was also used to evaluate the diagnostic accuracy to segregate the severe and non-severe groups of EASI, POEM, and pruritus-NRS. The box plots of the subjects divided by two groups for EASI, POEM, and pruritus-NRS are shown in Fig. S4-S5. Combinations of SCCA2 or LDH with other biomarkers were performed by the backward stepwise linear regression analysis and the results are shown in Fig S6. All statistical analyses were performed using Stata 17.0 (Stata Corp., College Station, TX, USA). The two-sided significance level for all tests was *P* < 0.05.

**References**

**Figure Legends**

**Table S1 Baseline characteristics of the subjects**

**Fig S1 Association of five biomarkers with EASI**

Dot plot analysis of the correlation between five biomarkers—IL-24, IL-25, IL-31, IL-33, and TSLP—and EASI in the subjects (n = 110) is depicted.

**Fig S2 Association of five biomarkers with POEM**

Dot plot analysis of the correlation between five biomarkers—IL-24, IL-25, IL-31, IL-33, and TSLP—and POEM in the subjects (n = 110) is depicted.

**Fig S3 Association of five biomarkers with pruritis-NRS**

Dot plot analysis of the correlation between five biomarkers—IL-24, IL-25, IL-31, IL-33, and TSLP—and pruritis NRS in the subjects (n = 110) is depicted.

**Fig S4 Biomarkers levels of the subjects divided into severe and non-severe groups for EASI**

The subjects are divided into severe and non-severe groups for EASI (≥ 21.1 or < 21.1) and the box plots between 14 biomarkers and these two groups are depicted.

**Fig S5 Biomarkers levels of the subjects divided into severe and non-severe groups for POEM**

The subjects are divided into severe and non-severe groups for POEM (≥ 17 or < 17) and the box plots between 14 biomarkers and these two groups are depicted.

**Fig S6 Biomarkers levels of the subjects divided into severe and non-severe groups for pruritus-NRS**

The subjects are divided into severe and non-severe groups for pruritus-NRS (≥ 4 or < 4) and the box plots between 14 biomarkers and these two groups are depicted.

**Fig S7 The ROC analysis of combinations of SCCA2 or LDH with other biomarkers by the stepwise analysis**

The ROC analyses of combinations of SCCA2 for EASI (**A**) or LDH for POEM (**B**) and pruritis-NRS (**C**) with other biomarkers were performed using the stepwise analysis and the AUCs of each combination are depicted.

References

1. Nakahara T, Izuhara K, Onozuka D, et al. Exploring biomarkers to predict clinical improvement of atopic dermatitis in patients treated with dupilumab (B-PAD study). Clin Exp Allergy. 2023; 53(2): 233-238.

2. Katayama I, Kohno Y, Akiyama K, et al. Japanese Guideline for Atopic Dermatitis 2014. Allergol Int. 2014; 63(3): 377-398.
