# Supplementary material for: The ability of biomarkers to assess the severity of atopic dermatitis
Source: J Allergy Clin Immunol Glob. 2023 Sep 27;3(1):100175. doi: 10.1016/j.jacig.2023.100175 (PMC10616407; doi:10.1016/j.jacig.2023.100175)
Supplement: Supplementary Table and Figures [file mmc2.pdf]

Table S1 Baseline characteristics of the subjects

	n=110	
Men, n (%)	74	(67.3)
Age (y), mean (SD)	40.3	(12.5)
Biomarker		
LDH (U/L)	264.8	(73.4)
Eosinophils (/μ L)	586.5	(506.9)
Total IgE (IU/mL)	11091.9	(9082.4)
SolubleIL-2R (U/mL)	416.7	(201.3)
CCL17/TARC (pg/mL)	2823.1	(4306.4)
CCL22/MDC (pg/mL)	1424.9	(1006.8)
CCL26/Eotaxin-3 (pg/mL)	43.1	(39.4)
IL-13 (pg/mL)	0.3	(0.2)
IL-22 (pg/mL)	8.5	(15.6)
CCL27/CTACK (pg/mL)	1087.2	(417.7)
CCL18/MIP-4/PARC (pg/mL)	127461.1	(99681.4)
ET1 (pg/mL)	1.1	(0.4)
Periostin (ng/mL)	103.7	(52.2)
SCCA2 (ng/mL)	8.3	(14.7)
Outcome		
EASI score, mean (SD)	26.6	(9.9)
POEM score, mean (SD)	18.2	(6.9)
pruritus-NRS, mean (SD)	6.5	(2.3)
uncomfortable skin-NRS, mean (SD)	6.3	(2.7)
treatment satisfaction-NRS, mean (SD)	5.1	(2.7)

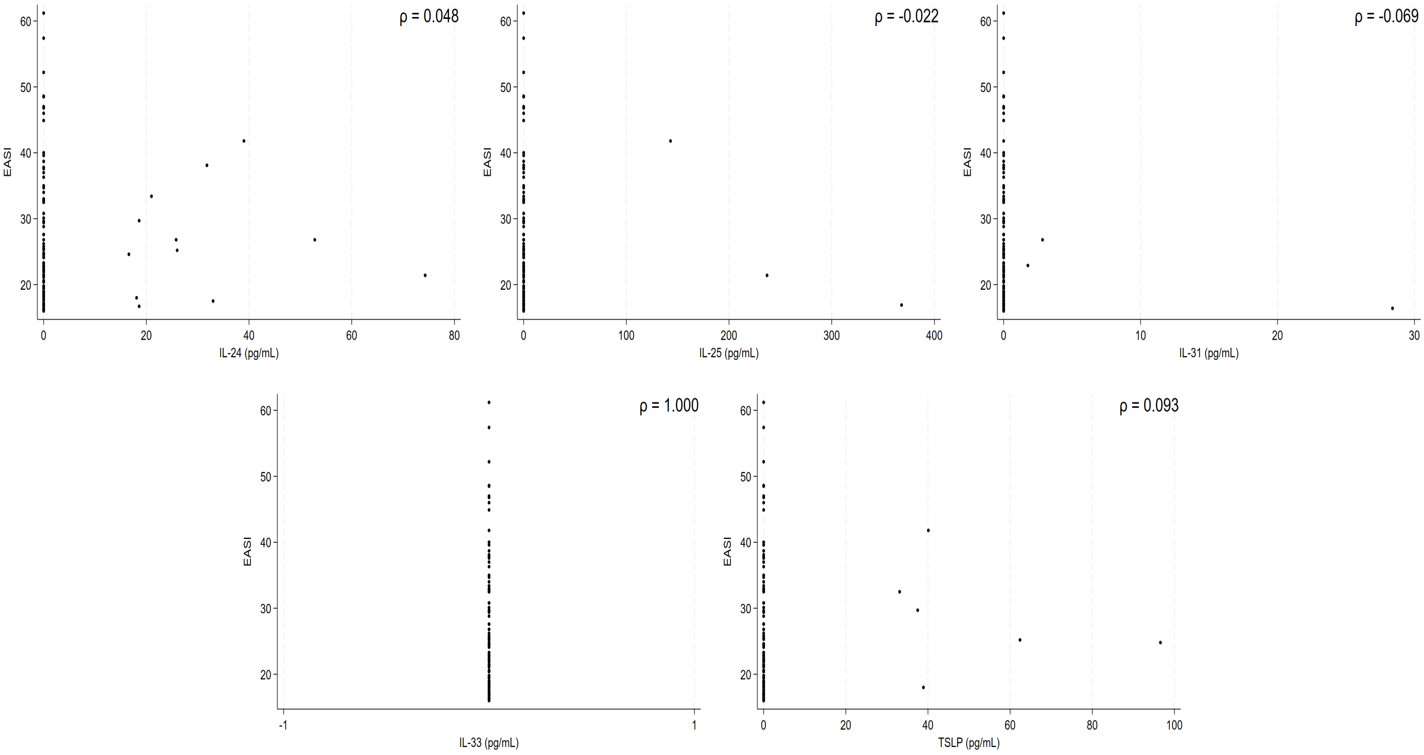
ρ = 0.048
EASI
IL-24 (pg/mL)
ρ = -0.022
EASI
IL-25 (pg/mL)
ρ = -0.069
EASI
IL-31 (pg/mL)
ρ = 1.000
EASI
IL-33 (pg/mL)
ρ = 0.093
EASI
TSLP (pg/mL)

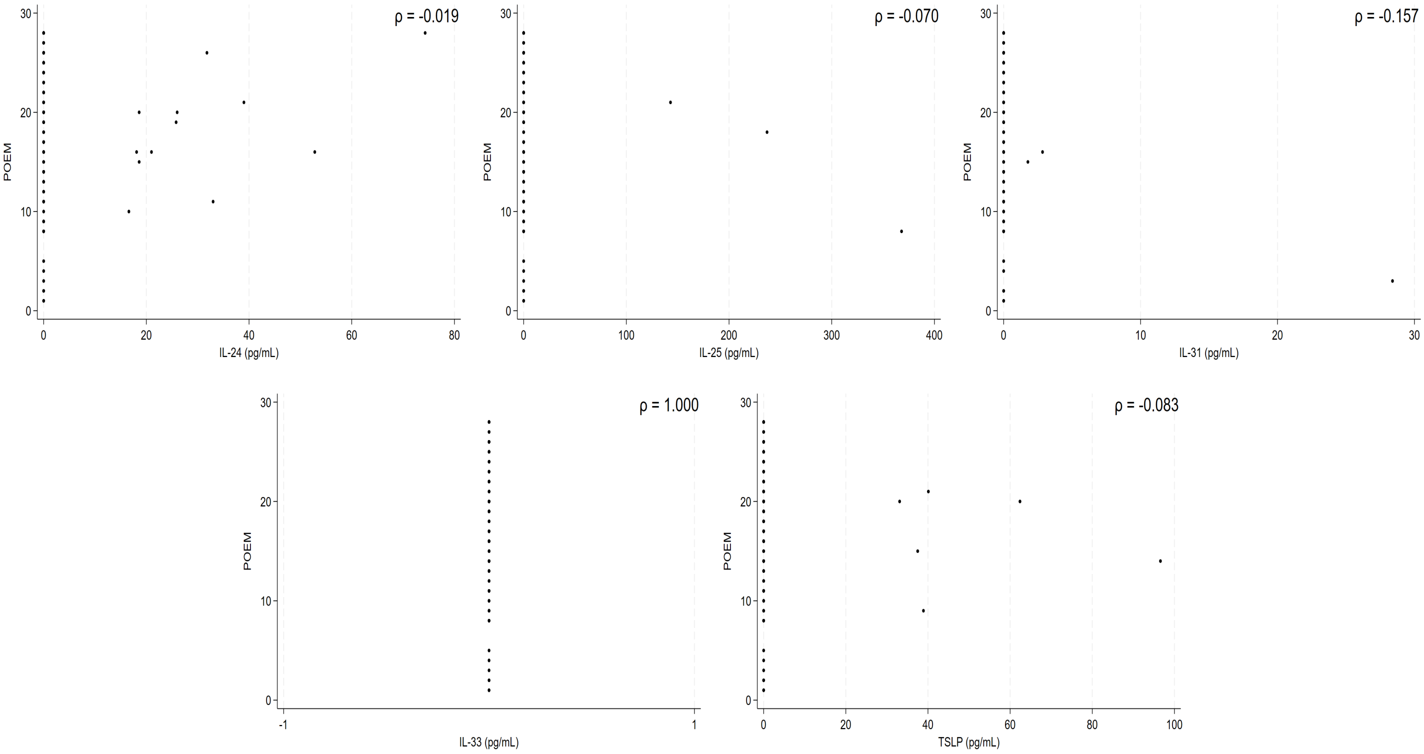
ρ = -0.019
POEM
IL-24 (pg/mL)
ρ = -0.070
POEM
IL-25 (pg/mL)
ρ = -0.157
POEM
IL-31 (pg/mL)
ρ = 1.000
POEM
IL-33 (pg/mL)
ρ = -0.083
POEM
TSLP (pg/mL)

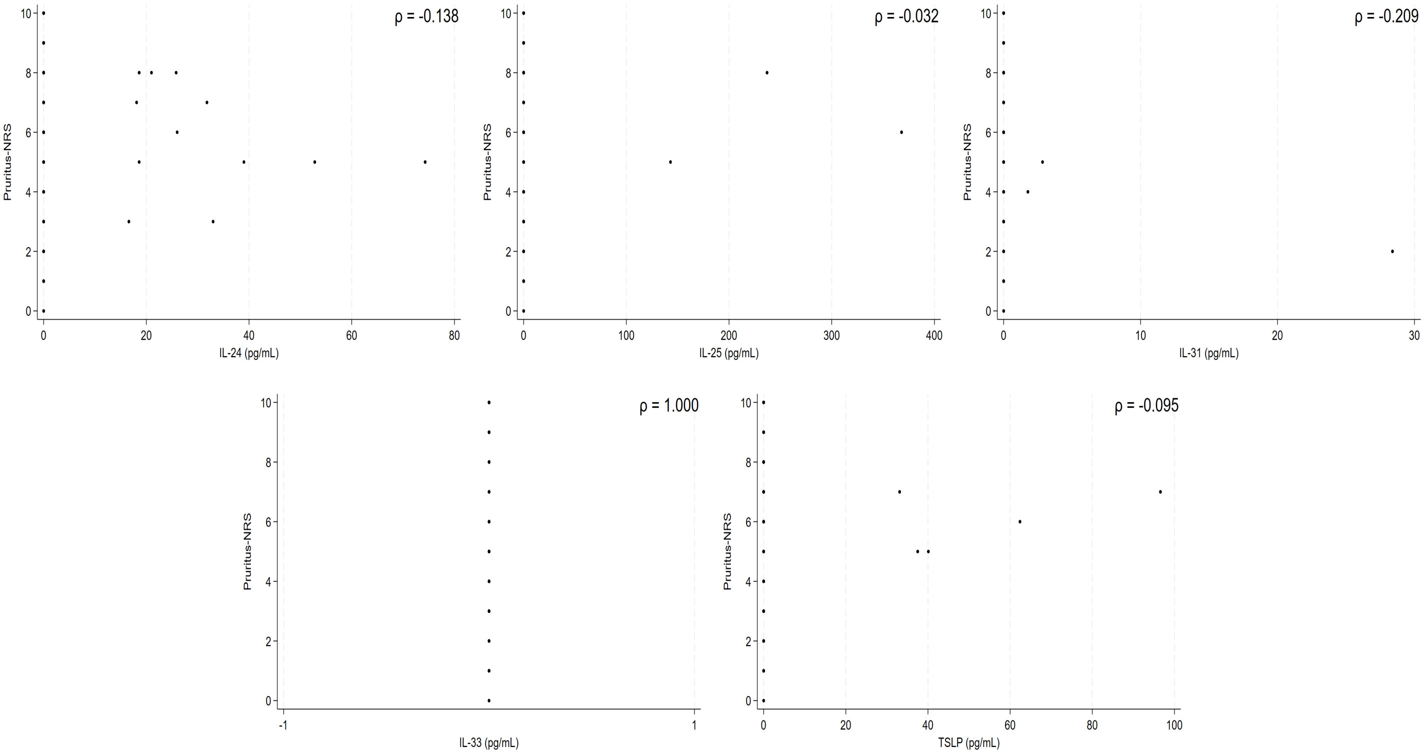
ρ = -0.138
Pruritus-NRS
IL-24 (pg/mL)
ρ = -0.032
Pruritus-NRS
IL-25 (pg/mL)
ρ = -0.209
Pruritus-NRS
IL-31 (pg/mL)
ρ = 1.000
Pruritus-NRS
IL-33 (pg/mL)
ρ = -0.095
Pruritus-NRS
TSLP (pg/mL)

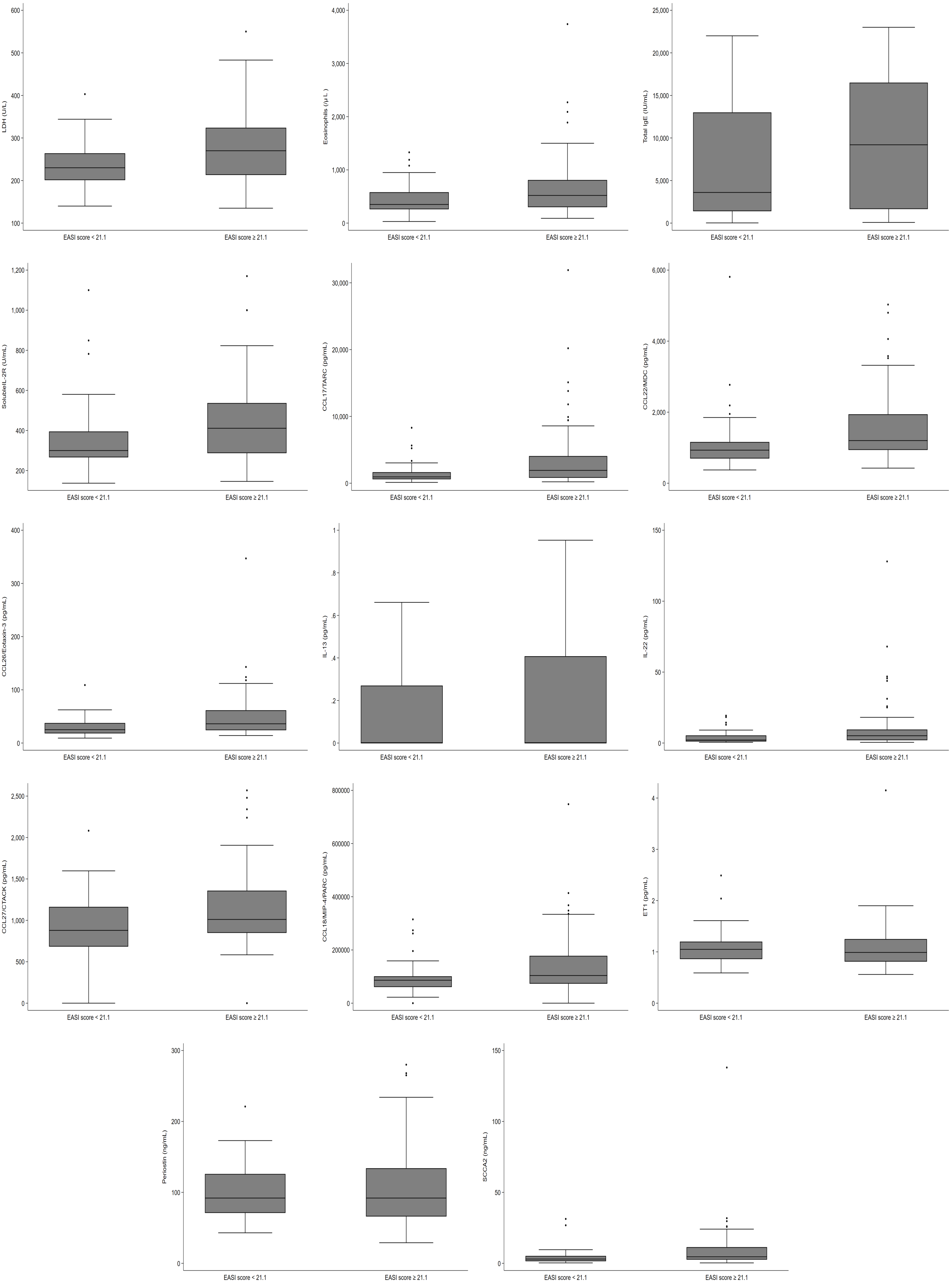
LDH (U/L)
Eosinophils (/μL)
Total IgE (IU/mL)
SolubleIL-2R (U/mL)
CCL17/TARC (pg/mL)
CCL22/MDC (pg/mL)
CCL26/Eotaxin-3 (pg/mL)
IL-13 (pg/mL)
IL-22 (pg/mL)
CCL27/CTACK (pg/mL)
CCL18/MIP-4/PARC (pg/mL)
ET1 (pg/mL)
Periostin (ng/mL)
SCCA2 (ng/mL)
EASI score < 21.1
EASI score ≥ 21.1

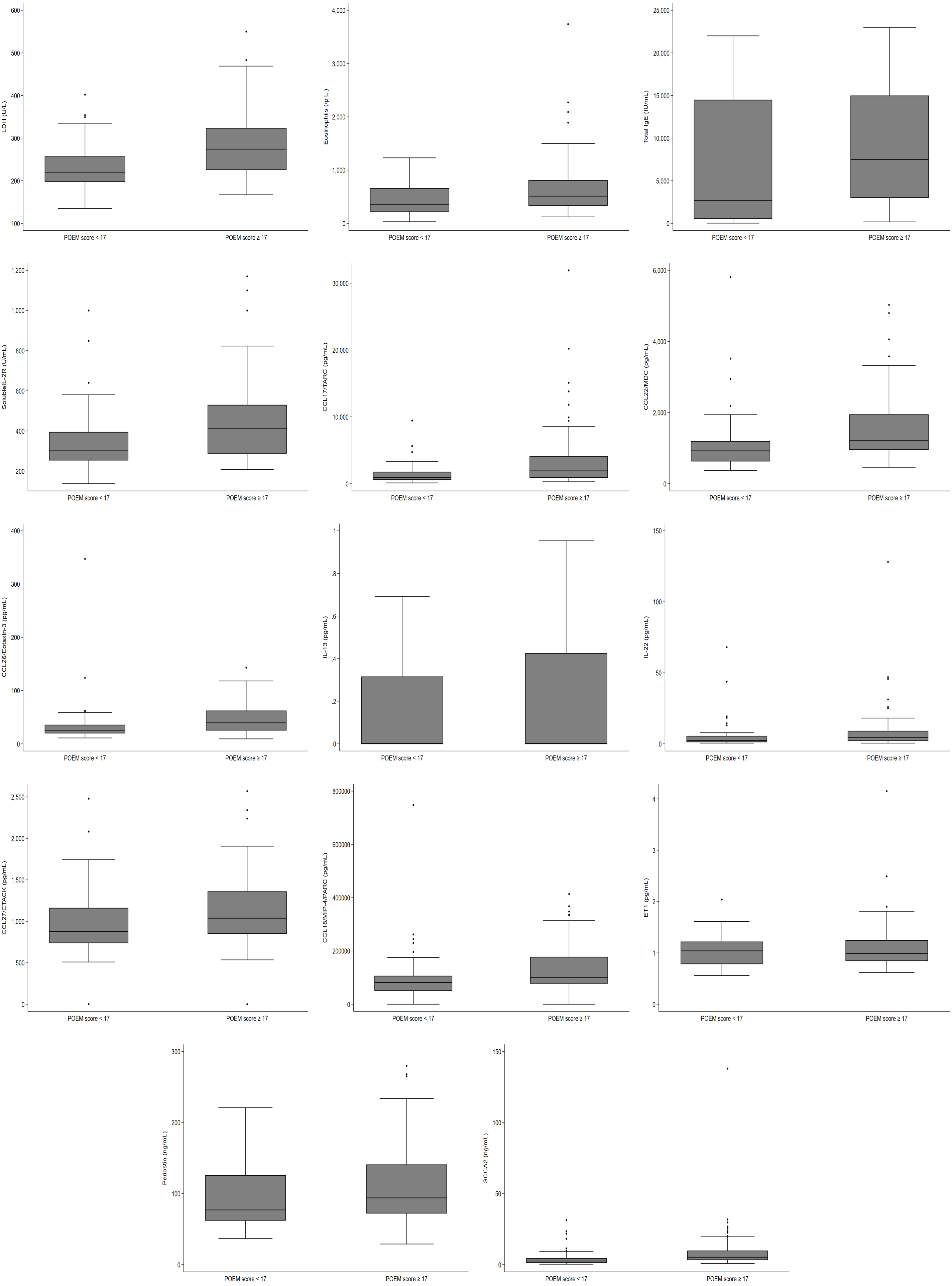
LDH (U/L)
Eosinophils (/μL)
Total IgE (IU/mL)
SolubleIL-2R (U/mL)
CCL17/TARC (pg/mL)
CCL22/MDC (pg/mL)
CCL26/Eotaxin-3 (pg/mL)
IL-13 (pg/mL)
IL-22 (pg/mL)
CCL27/CTACK (pg/mL)
CCL18/MIP-4/PARC (pg/mL)
ET1 (pg/mL)
Periostin (ng/mL)
SCCA2 (ng/mL)
POEM score < 17
POEM score ≥ 17

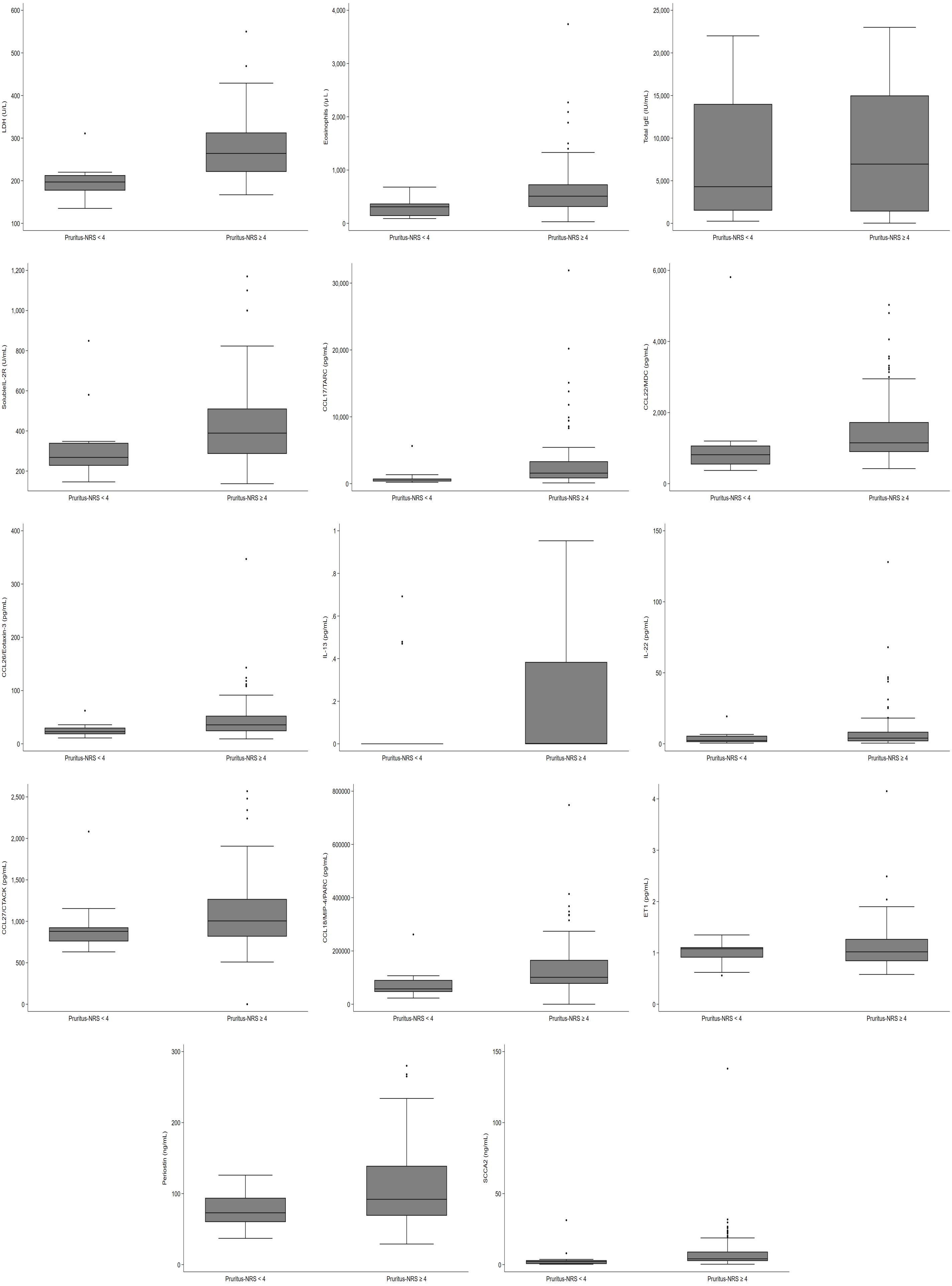
LDH (U/L)
Eosinophils (/μ L)
Total IgE (IU/mL)
SolubleIL-2R (U/mL)
CCL17/TARC (pg/mL)
CCL22/MDC (pg/mL)
CCL26/Eotaxin-3 (pg/mL)
IL-13 (pg/mL)
IL-22 (pg/mL)
CCL27/CTACK (pg/mL)
CCL18/MIP-4/PARC (pg/mL)
ET1 (pg/mL)
Periostin (ng/mL)
SCCA2 (ng/mL)
Pruritus-NRS < 4
Pruritus-NRS ≥ 4

A

EASI score ≥ 21.1 vs. EASI score < 21.1	
Combination	AUC
SCCA2, Eosinophils , Total IgE, SolubleIL-2R , CCL17/TARC, CCL22/MDC	0.662
SCCA2, Eosinophils , Total IgE, SolubleIL-2R , CCL17/TARC	0.642
SCCA2, Eosinophils , Total IgE, SolubleIL-2R	0.651
SCCA2, Eosinophils , Total IgE	0.674
SCCA2, Eosinophils	0.680

B

POEM score ≥ 17 vs. POEM score < 17	
Combination	AUC
LDH, IL-22	0.710

C

pruritis-NRS score ≥ 4 vs. pruritis-NRS score < 4	
Combination	AUC
LDH, IL-22, CCL26/Eotaxin-3, CCL18/MIP-4/PARC	0.877
LDH, IL-22, CCL26/Eotaxin-3	0.867
LDH, IL-22	0.858
